# Letter to the Editor: Chikungunya Virus Infection—An Update on Chronic Rheumatism in Latin America

**DOI:** 10.5041/RMMJ.10288

**Published:** 2017-01-30

**Authors:** Alfonso J. Rodriguez-Morales

**Affiliations:** Public Health and Infection Research Group, Faculty of Health Sciences, Universidad Tecnológica de Pereira, Pereira, Risaralda, Colombia; and Colombian Collaborative Network on Zika and Other Arboviruses (RECOLZIKA), Pereira, Risaralda, Colombia

**To the Editor,**

The article of Krutikov and Manson^1^ was interesting. However, no comment was made on the impact and related clinical epidemiology of the chikungunya virus (CHIKV) infection during the 2014–2015 epidemics in Latin America, the most recent area affected by CHIKV. Certainly, persistent musculoskeletal manifestations of the disease have been shown to affect a highly variable proportion of infected patients (even >87%). Following the epidemics in La Réunion Island and India,^2^ and now in Latin America, this disease is having a significant impact.

That impact should be obvious when considering the officially reported cases: over 2 million were noted in the Americas between 2014 and 2016.^3^ Nevertheless, in countries such as Colombia (with over 1 million cases officially reported), final estimations indicate that over 3 million cases of CHIKV infection were detected. During 2014, as epidemics began in the region, initial estimates of the potential impact were based primarily on the experience in La Réunion and India. These data provided an expected prevalence of 47.57% for post-chikungunya chronic inflammatory rheumatism (pCHIKV-CIR) (95% CI 45.08%–50.13%).^4^

In Colombia, the Sucre department (state)^5^ published a first study in the Americas in August 2015 with information about a small cohort (*n*=39) that was followed for a median of 37 weeks; 89.7% (95% CI 75.8%–97.1%) of those followed developed pCHIKV-CIR. Also in August 2015, Colombia’s Tolima department provided data on a larger study (*n*=131). Over a 24-week follow-up period the pCHIKV-CIR prevalence was 44.3% (95%CI 35.39%–53.16%), being higher in women over 40 years of age (52.3, 95% CI 36.38%–68.17%).^6^ In March 2016, an even larger Latin American study (*n*=283), from Colombia’s Risaralda department, was published showing that 53.7% (95% CI 47.7%–59.7%) of patients presented with pCHIKV-CIR in a median time of 14.6 weeks.^7^ By pooling together the data from studies in Colombia, a combined prevalence of pCHIKV-CIR of 56.6% is obtained (95% CI 40.5%–72.6%; *n*=453; *I^2^*=75.3; *Q*=8.1).

Hence, this disease can last for months and years, and presents important concerns about the associated disability (estimated as disability-adjusted life years (DALYs) lost), which have already been assessed in Latin America. Countries such as the Dominican Republic, El Salvador, Puerto Rico, and Colombia are among the most affected (>30 DALYs lost per 100,000 population).^8^,^9^ In 2014 alone, Colombia estimated that the cost of CHIKV infection to the country will be in the magnitude of 73.6 million dollars, up to 185.6 million dollars,^10^ with regions showing >2,800 DALYs lost per 100,000 population (San Juan de Nepomuceno, Bolivar).^10^

Many questions remain on the multiple clinical aspects of pCHIKV-CIR. As Krutikov and Manson^1^ already stated, the etiology of this arthralgia is not fully understood and requires more research. Undoubtedly, CHIKV has become a major threat, particularly in Latin America, where—despite efforts to control the disease better—transmission still occurs, with more than 300,000 new cases detected in the region in 2016.

## Abbreviations

CHIKV: chikungunya virus
pCHIKV-CIR: post-chikungunya chronic inflammatory rheumatism
